# Timing Specific Requirement of microRNA Function is Essential for Embryonic and Postnatal Hippocampal Development

**DOI:** 10.1371/journal.pone.0026000

**Published:** 2011-10-04

**Authors:** Qingsong Li, Shan Bian, Janet Hong, Yoko Kawase-Koga, Edwin Zhu, Yongri Zheng, Lizhuang Yang, Tao Sun

**Affiliations:** 1 Department of Cell and Developmental Biology, Weill Medical College, Cornell University, New York, New York, United States of America; 2 The 2nd Affiliated Hospital, Harbin Medical University, Harbin, Heilongjiang, China; 3 Department of Oral and Maxillofacial Surgery, The University of Tokyo Hospital, Tokyo, Japan; 4 Department of Biology, New York University, New York, New York, United States of America; Université Pierre et Marie Curie-Paris6, INSERM, CNRS, France

## Abstract

The adult hippocampus consists of the dentate gyrus (DG) and the CA1, CA2 and CA3 regions and is essential for learning and memory functions. During embryonic development, hippocampal neurons are derived from hippocampal neuroepithelial cells and dentate granular progenitors. The molecular mechanisms that control hippocampal progenitor proliferation and differentiation are not well understood. Here we show that noncoding microRNAs (miRNAs) are essential for early hippocampal development in mice. Conditionally ablating the RNAase III enzyme *Dicer* at different embryonic time points utilizing three *Cre* mouse lines causes abnormal hippocampal morphology and affects the number of hippocampal progenitors due to altered proliferation and increased apoptosis. Lack of miRNAs at earlier stages causes early differentiation of hippocampal neurons, in particular in the CA1 and DG regions. Lack of miRNAs at a later stage specifically affects neuronal production in the CA3 region. Our results reveal a timing requirement of miRNAs for the formation of specific hippocampal regions, with the CA1 and DG developmentally hindered by an early loss of miRNAs and the CA3 region to a late loss of miRNAs. Collectively, our studies indicate the importance of the Dicer-mediated miRNA pathway in hippocampal development and functions.

## Introduction

The hippocampus is a well studied brain structure due to its ability to process learning and memory functions. The adult hippocampus is located in the caudomedial edge of the mouse neocortex with a well-defined “C” shape structure that consists of the CA1, CA2, CA3 and the dentate gyrus (DG) regions. The subgranular zone (SGZ) in the adult hippocampal DG contains neural stem cells (NSCs), which can self-renew and give rise to both neurons and glia [1], [2]. The presence of active neurogenesis in the adult hippocampus is likely related to cognitive functions and highlights adult NSCs as a promising means for stem cell based therapy for neurodegeneration disorders [3], [4].

During brain development, the hippocampus is derived from the medial pallial domain adjacent to the cortical hem in the telencephalon [5], [6]. The developing DG has distinct morphology compared to the hippocampal neuroepithelium and consists of migratory granular progenitors [5], [6]. The cortical hem is a signaling center that releases multiple signaling molecules, which play an essential role in hippocampal development [7]–[11]. For example, *Wnt3a* is expressed in the cortical hem and its downstream gene *Lef1* is expressed in the hippocampus. Previous studies have shown that *Wnt3a* and *Lef1* mutant mice display severe hippocampal defects [12], [13]. Transcription factors, such as Emx2, expressed in hippocampal neuroepithelial cells; and Lhx5, expressed in the cortical hem, are also important for morphogenesis of the developing hippocampus [14]–[16]. However, the molecular mechanisms that control precise and dynamic expression of the molecules essential for hippocampal development remain unclear.

A new post-transcriptional gene regulation mediated by microRNAs (miRNAs) has been demonstrated to have an important role in the development of the central nervous system (CNS). MiRNAs are approximately 22 nucleotide (nt) endogenous noncoding small RNAs [17], [18]. MiRNA precursors are processed into mature miRNAs by the RNAase III enzyme Dicer [19], [20]. A mature miRNA recognizes a complementary sequence in the 3′-untranslated region (3′-UTR) of its target messenger RNA (mRNA) and affects mRNA stability and/or silences protein translation [21], [22].

Because miRNAs are processed by Dicer, genetic ablation of *Dicer* in specific tissues has revealed a global functional role of miRNAs in development. Regional specific deletions of *Dicer* expression in the CNS using different *Cre* lines result in smaller cortical size and abnormal development of the midbrain and spinal cord [23]–[25]. Under a culture condition, cortical NSCs lacking *Dicer* also show survival defects and abnormal differentiation [26], [27]. *Dicer* ablation in postmitotic neurons using the *CaMKII-Cre* line results in a smaller cortex and impaired dendritic branching in pyramidal neurons in the CA1 region [28], [29]. Interestingly, mice with *Dicer* ablation in the adult brain using inducible *CaMKII- CreERT2* line show enhanced learning and memory [30]. These studies, particularly the different timings of *Dicer* deletion, imply an important role of miRNAs in brain development, especially in hippocampal morphogenesis and functions.

To reveal miRNA functions in embryonic and postnatal hippocampal development, we here have examined hippocampal neurogenesis and formation by ablating *Dicer* expression in the brain at different embryonic time points using three *Cre* lines. We show that miRNAs are required for early proliferation of both hippocampal neuroepithelial cells and dentate granular progenitors. The differentiation of pyramidal neurons in the CA1 and CA3 regions and granule cells in the dentate gyrus is also affected by the different timings of the loss of miRNAs. Our results indicate an important temporal function of miRNAs in modulating morphogenesis of distinct regions in the developing hippocampus.

## Results

### miRNA function is required for normal hippocampal development

**Figure 1:**
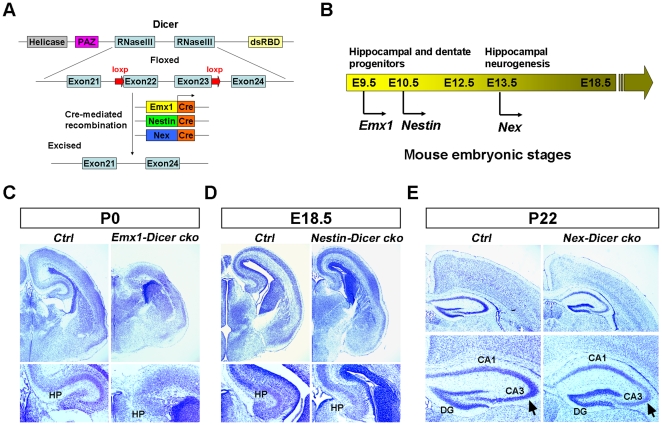
Conditional deletion of *Dicer* causes abnormal hippocampal development. (A) The targeting strategy for *Dicer* conditional deletions in the central nervous system using three *Cre* lines. The N-terminal RNA helicase domain, PAZ domain, two Ribonuclease III (RNaseIII) domains as well as a double-stranded RNA-binding domain (dsRBD) are labeled. The Exon22 and Exon23 of *Dicer* are conditionally excised by *Cre* lines. (B) *Emx1-Cre*, *Nestin-Cre* and *Nex-Cre* are active at different stages of hippocampal development in mouse embryos. (C) The hippocampus (HP) of *Emx1-Dicer* conditional knockout (*Emx1-Dicer cko*) mice displayed abnormal morphology compared to the control (*Ctrl*) at postnatal day 0 (P0), as detected by Nissl staining on coronal sections. (D) In embryonic day 18.5 (E18.5) *Nestin-Dicer* conditional knockout (*Nestin-Dicer cko*) mice, the hippocampus was smaller than controls. (E) The hippocampus of *Nex-Dicer* conditional knockout (*Nex-Dicer cko*) mice was slightly smaller than controls. While the CA1 and dentate gyrus (DG) did not change, the CA3 region (arrows) was thinner in the *Nex-Dicer cko* hippocampus than controls.

To examine whether miRNAs play a role in hippocampal development, we applied mouse genetic tools to ablate Dicer expression in developing brains using a *Cre-loxp* system. Floxed *Dicer* mice (*Dicer^loxp/loxp^*) with two *loxP* sites flanking exon 22 and exon 23, which encode the RNAase III domains of *Dicer*, were bred with three *Cre* lines: *Emx1-Cre*, *Nestin-Cre* and *Nex-Cre* lines (Fig. 1A). All three *Cre* lines are active in the developing hippocampus but commence at different time points, with *Emx1-Cre* active by embryonic day 9.5 (E9.5) and *Nestin-Cre* by E10.5 [31]–[33]. *Nex-Cre* line displays activity in the hippocampus by E13.5 [34]. Utilization of the *Emx1-Cre* and *Nestin-Cre* lines will ablate most progenitors, while the *Nex-Cre* line will affect postmitotic neurons. By utilizing these three *Cre* lines, we were able to examine temporal functions of miRNAs in hippocampal progenitor development and neurogenesis at different embryonic stages (Fig. 1B).

Mice with *Dicer* ablation using the *Emx1-Cre* line (called *Emx1-Dicer cko*) displayed a smaller cortex and strikingly, the morphology of the hippocampus (HP) was not clearly distinguishable at postnatal day 0 (P0) (Fig. 1C). Mice with *Dicer* ablation using the *Nestin-Cre* line (called *Nestin-Dicer cko*) died after birth [24]. In E18.5 brains, hippocampal morphogenesis occurred normally but the size was reduced, while the whole brain did not change in size significantly in *Nestin-Dicer cko* mice (Fig. 1D). Later ablation of *Dicer* using the *Nex-Cre* line (called *Nex-Dicer cko*) did not significantly affect hippocampal morphology. However, *Nex-Dicer cko* mice had a smaller brain compared to control litter mates at P22, and noticeably, the CA3 region in the hippocampus was thinner (Fig. 1E). These results indicate that miRNA functions are required for normal hippocampal development. The more severe defect of the *Emx1-Dicer cko* hippocampus suggests that earlier *Dicer* ablation may primarily affect hippocampal progenitor development.

### Dicer deletion blocks miRNA biogenesis in the developing hippocampus

**Figure 2:**
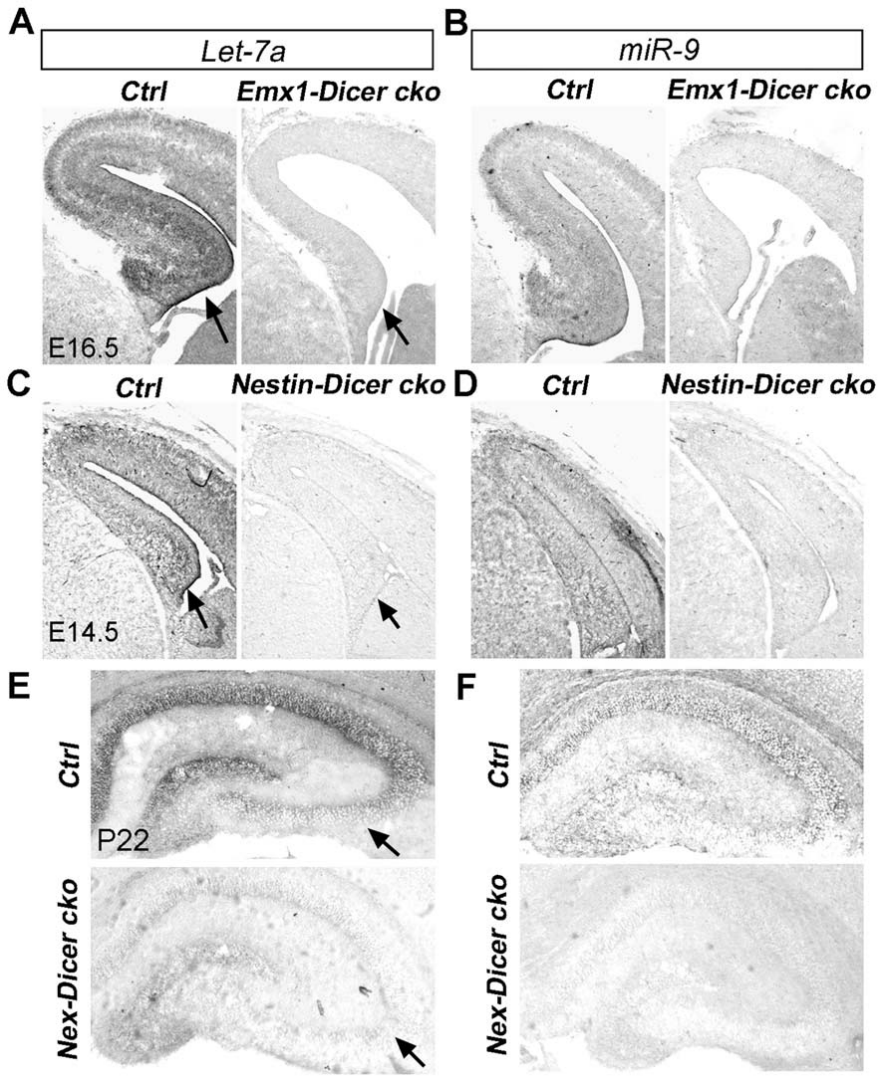
Reduction of miRNA expression levels in *Dicer* conditional knockout hippocampus. (A, C, E) Compared to the control (*Ctrl*) brains, expression of *Let-7a* in the hippocampus (arrows) was undetectable in E16.5 *Emx1-Dicer cko* and E14.5 *Nestin-Dicer cko* brains, and was significantly reduced in P22 *Nex-Dicer cko* brains, as detected by *in situ* hybridization. (B, D, F) Expression of *miR-9* was greatly reduced in the hippocampus of E16.5 *Emx1-Dicer cko*, E14.5 *Nestin-Dicer cko*, and P22 *Nex-Dicer cko* mouse brains.

To demonstrate whether abnormal hippocampal development was caused by the absence of miRNA biogenesis, we examined expression of representative miRNAs in *Dicer* conditional knockout brains using *in situ* hybridization. In E16.5 control mice, while miRNAs *Let-7a* and *miR-9* were highly expressed in the cortex and the hippocampus, their expression was undetectable in *Emx1-Dicer cko* brains (Fig. 2A, B). Similarly, *Let-7a* and *miR-9* expression was depleted in the E14.5 *Nestin-Dicer cko* cortex and hippocampus, and was greatly reduced in the P22 *Nex-Dicer cko* hippocampus (Fig. 2C–F). Our results indicate that miRNA biogenesis is significantly blocked in the hippocampus of *Dicer* conditional knockout mice generated using all three *Cre* lines, confirming that the perceived hippocampal defects are mostly caused by the loss of miRNAs.

### Dicer deletion affects expression patterns of early hippocampal markers

**Figure 3:**
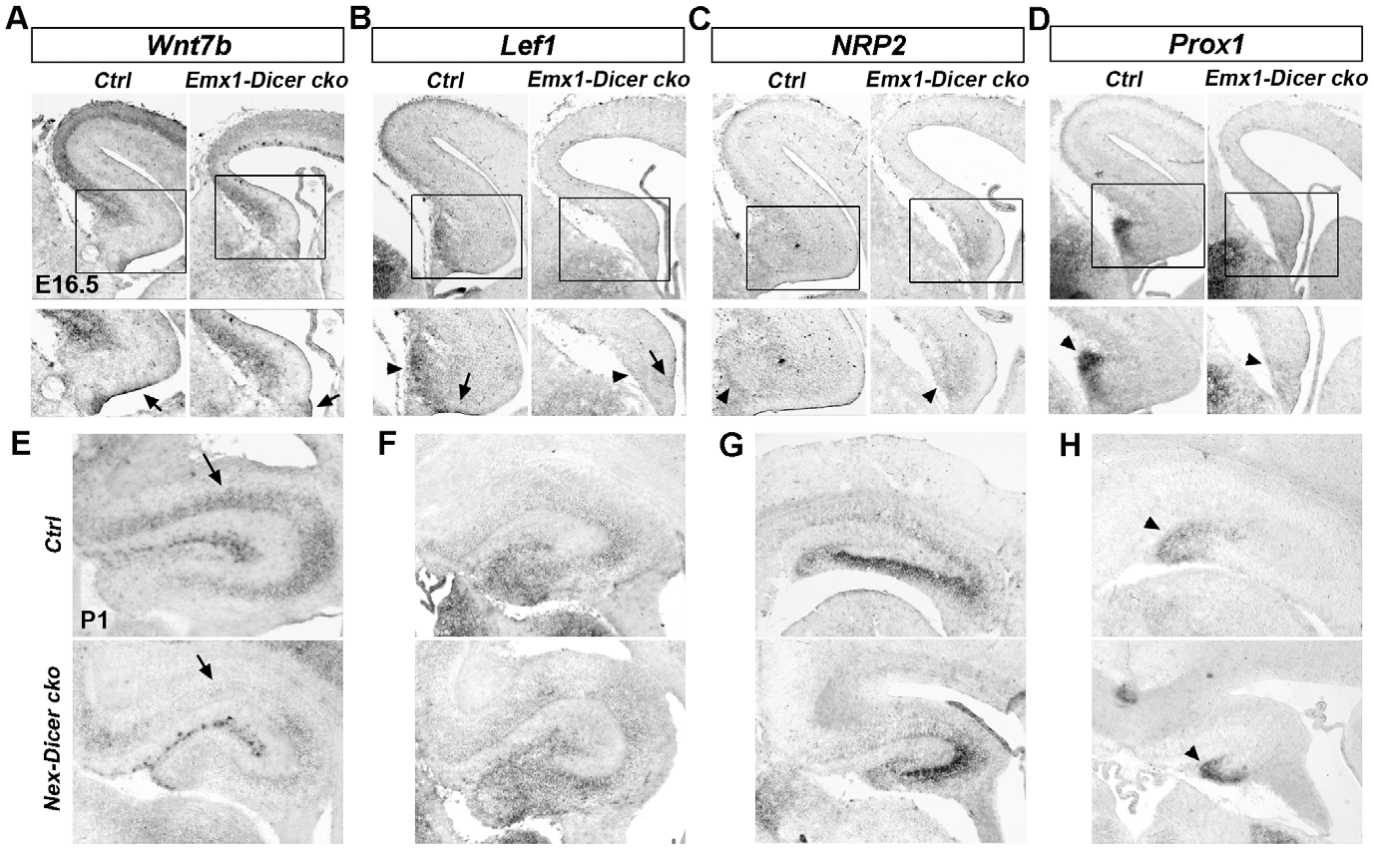
Abnormal expression patterns of hippocampal markers in *Dicer* conditional knockout mouse brains. (A, B) *Wnt7b* expression in the fimbria region adjacent to the hippocampus (arrows) and *Lef1* expression in the entire hippocampus (arrowheads) were reduced in the E16.5 *Emx1-Dicer cko* hippocampus compared to controls (*Ctrl*). (C, D) *Neuropilin 2* (*NRP2*) expression in the hippocampus did not change significantly, while *Prox1* expression in the dentate gyrus (DG, arrowheads) was greatly reduced in the *Emx1-Dicer cko* hippocampus. (E–H) In the P1 *Nex-Dicer cko* hippocampus, while *Wnt7b* expression was reduced (arrows), expression of *Lef1*, *NRP2* and *Prox1* did not change significantly. Note that the DG was smaller in the *Nex-Dicer cko* hippocampus (arrowheads).

We next examined whether hippocampal morphogenesis defects are caused by altered expression of hippocampal markers [5], [6]. In the developing brain, *Wnt7b* is expressed in the fimbria region adjacent to the hippocampus, while Wnt downstream gene *Lef1* is expressed in the entire hippocampus [12]. In the E16.5 *Emx1-Dicer cko* fimbria region and hippocampus, where miRNA biogenesis was blocked at an early stage, expression of *Wnt7b* and *Lef1* was greatly reduced compared to controls (Fig. 3A, B). In the wild type hippocampus, while *Neuropilin 2* (*NRP2*) is expressed in the entire hippocampus, homeobox gene *Prox1* is expressed highly in the DG [12]. In the E16.5 *Emx1-Dicer cko* hippocampus, *NRP2* expression did not display significant changes, but *Prox1* expression was greatly reduced in the DG (Fig. 3C, D). Similarly, in the P1 *Nex-Dicer cko* hippocampus, where miRNA biogenesis was blocked at a late stage, *Wnt7b* expression was reduced, but *Lef1* and *NRP2* expression did not change significantly (Fig. 3E-G). Even though the DG was smaller in the *Nex-Dicer cko* hippocampus, the intensity of *Prox1* expression in the DG was not changed (Fig. 3H).

**Figure 4:**
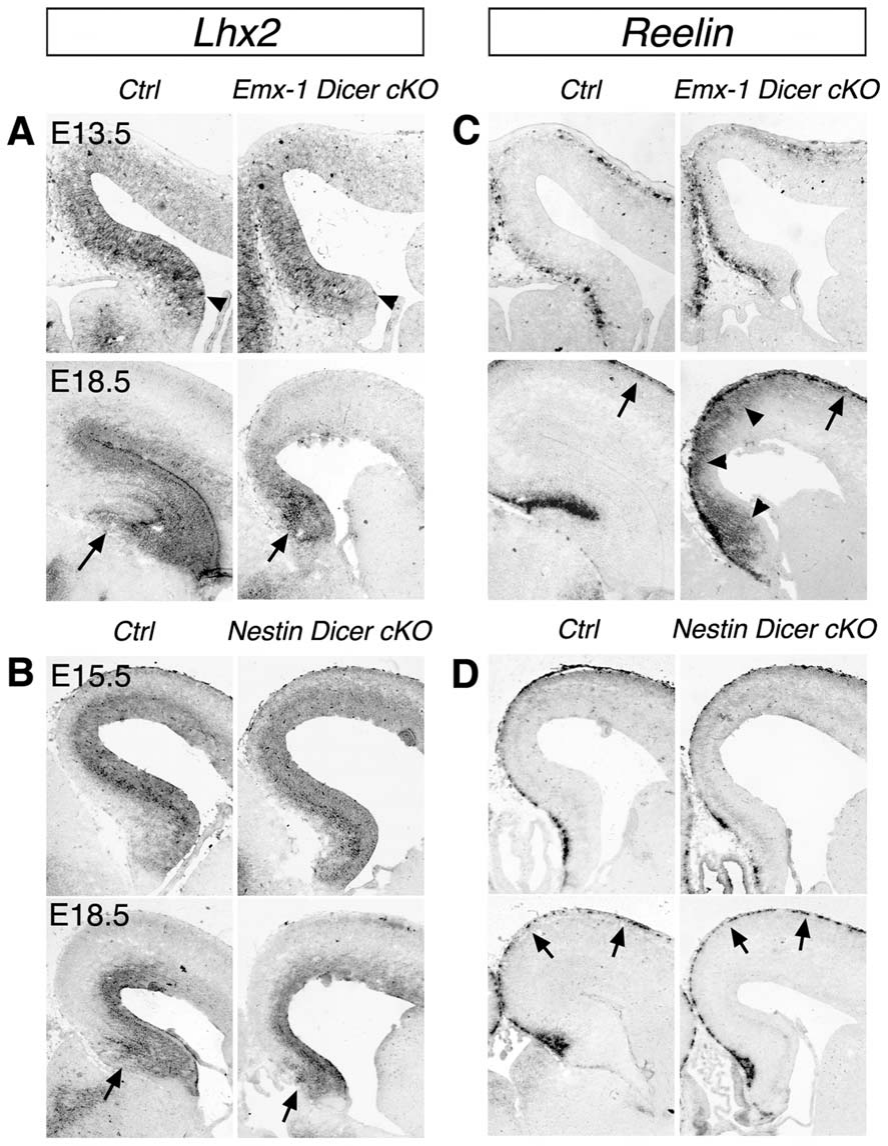
Abnormal production and migration of Cajal-Retzius neurons in *Dicer* conditional knockout mouse brains. (A, B) *Lhx2* expression was reduced in the E13.5 (arrowheads) and E18.5 (arrows) hippocampus in *Emx1-Dicer cko* and *Nestin-Dicer cko* mice. (C, D) *Reelin* expression, which labels Cajal-Retzius neurons (arrows), was normal in the E13.5 *Emx1-Dicer cko* cortex, and E15.5 and E18.5 *Nestin-Dicer cko* cortices. However, ectopic *Reelin* expression was detected in the E18.5 *Emx1-Dicer cko* cortex (arrowheads).

We then examined expression of transcription factor Lhx2, which is expressed in the early hippocampus [5]. *Lhx2* expression was reduced in the E13.5 *Emx1-Dicer cko* hippocampus. The reduction of *Lhx2* expression was more profound in the E18.5 *Emx1-Dicer cko* mice, which exhibited a significantly reduced hippocampus size (Fig. 4A). Similarly, *Lhx2* expression was reduced in the E18.5 *Nestin-Dicer cko* hippocampus (Fig. 4B). Previous work has shown that reduced Lhx2 expression causes an expansion of the cortical hem, where Cajal-Retzius (CR) neurons are generated [8], [35]. We thus examined expression of Reelin, a CR neuron marker. While *Reelin* expression did not show significant changes in the E13.5 *Emx1-Dicer cko* cortex, ectopic *Reelin* expression was detected in the E18.5 *Emx1-Dicer cko* cortex (Fig. 4C). However, *Reelin* expression was normal in the E15.5 and E18.5 *Nestin-Dicer cko* cortex (Fig. 4D).

Our results indicate that early depletion of *Dicer* expression affects expression patterns of hippocampal markers, which may contribute to hippocampal morphogenesis defects. Moreover, altered expression of early markers in the hippocampus perhaps affects cortical hem development and results in abnormal production and migration of CR neurons in the cortex.

### Loss of miRNAs reduces early hippocampal and dentate granular progenitor numbers

**Figure 5:**
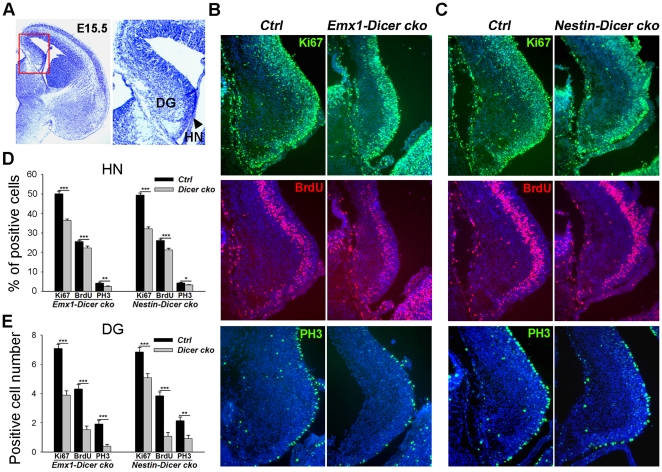
Early *Dicer* deletion in the brain affects proliferation of hippocampal and dentate granular progenitors. (A) A coronal section with Nissl staining of E15.5 wild type mouse brains. The highlighted region is the developing hippocampus. The hippocampal neuroepithelium (HN, arrow) and dentate gyrus (DG) are labeled. (B, C) Numbers of progenitors in the HN and DG regions were greatly reduced when *Dicer* was ablated in early developing brains. Progenitors were detected with anti-Ki67, anti-BrdU, and anti-PH3 antibodies in E15.5 *Emx1-Dicer cko*, *Nestin-Dicer cko* and control (*Ctrl*) brains. (D, E) Quantification of Ki67^+^, BrdU^+^ and PH3^+^ cells in the E15.5 HN and DG regions. At least three *Emx1-Dicer cko*, *Nestin-Dicer cko* and three control animals were used for all statistical analysis. n>3, *, *P<0.05*; **, *P<0.01*; ***, *P<0.001*.

In the developing hippocampus, neurons in the CA1 and CA3 regions are derived from embryonic hippocampal neuroepithelial cells [5], [6]. The dentate granular cells are derived from the presumptive dentate gyrus (DG) region adjacent to the hippocampal neuroepithelium (HN) (Fig. 5A). To further examine whether abnormal hippocampal morphology in *Dicer* knockout brains was caused by defects in progenitors, we examined proliferation and survival of hippocampal progenitors.

Ki67 labels most progenitor cells in the G1, S, G2 and M-phase. We detected a great reduction of Ki67^+^ cell numbers in the HN region in both *Emx1-Dicer cko* and *Nestin-Dicer cko* brains at E15.5 (Fig. 5B-D). After a 30 min pulse of BrdU injection, which labels cells in the S-phase of the cell cycle, we detected fewer BrdU^+^ cells in the *Dicer* knockout HN (Fig. 5B–D). Moreover, the number of cells positive for phospho-histone H3 (PH3), which labels mitotic cells in the M-phase of the cell cycle, was greatly reduced in the HN of E15.5 *Emx1-Dicer cko* and *Nestin-Dicer cko* hippocampus (Fig. 5B–D).

Further analysis of dentate granular progenitor development revealed that similarly to the HN, numbers of Ki67^+^, BrdU^+^ and PH3^+^ cells were greatly reduced in the DG region when *Dicer* expression was deleted in *Emx1-Dicer cko* and *Nestin-Dicer cko* brains (Fig. 5B–E). Thus, the early loss of miRNA biogenesis using *Emx1-Cre* and *Nestin-Cre* lines causes a significant reduction of proliferating hippocampal neuroepithelial cells and dentate granular progenitors.

### Dicer deletion causes a continuous loss of hippocampal progenitors

**Figure 6:**
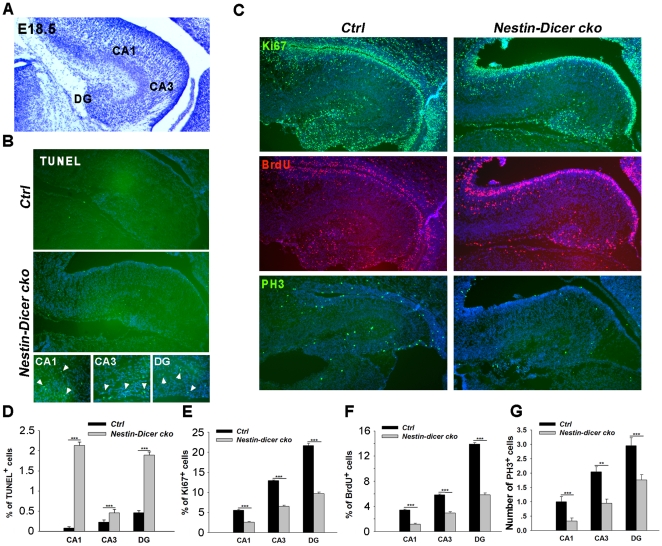
Increased cell death and decreased proliferation of neural progenitors in the E18.5 *Nestin-Dicer cko* hippocampus. (A) A coronal section with Nissl staining of the E18.5 wild type hippocampus. The CA1, CA3, and dentate gyrus (DG) regions are labeled. (B) Increased apoptotic cells in the hippocampus of *Nestin-Dicer cko* brains (arrowheads) compared to controls (*Ctrl*), as detected by TUNEL assays. (C) The number of hippocampal progenitors was decreased in *Nestin-Dicer cko* brains. Progenitors were detected with anti-Ki67, anti-BrdU, and anti-PH3 antibodies. (D-G) Quantification of TUNEL^+^, Ki67^+^, BrdU^+^ and PH3^+^ cells in the *Nestin-Dicer cko* and control hippocampus. At least three *Nestin-Dicer cko* and three control animals were used for all statistical analysis. n>3, **, *P<0.01*; ***, *P<0.001*.

The CA1, CA3 and DG regions in the hippocampus are mostly formed at E18.5 (Fig. 6A). To test the continuous effects of *Dicer* deletion in hippocampal progenitor development, we examined the proliferation and survival of progenitor cells in the E18.5 hippocampus. Because the hippocampal morphology was severely disrupted in *Emx1-Dicer cko* brains by E18.5, mostly due to reduced proliferation and increased cell death (data not shown), the *Nestin-Dicer cko* mice were analyzed.

Using a TUNEL assay to detect apoptotic cells, we observed an increase of TUNEL^+^ cells in the E18.5 *Nestin-Dicer cko* hippocampus, particularly in the CA1 and DG regions (Fig. 6B, D). Moreover, we detected decreased numbers of BrdU^+^ and Ki67^+^ cells in the CA1, CA3 and DG regions when *Dicer* was deleted (Fig. 6C–G). Our analyses suggest that the smaller hippocampus in E18.5 *Nestin-Dicer cko* brains is caused by a decrease of proliferating hippocampal and dentate granular progenitors, and an increase of apoptotic cells.

### Dicer deletion causes early differentiation and abnormal maturation of hippocampal neurons

**Figure 7:**
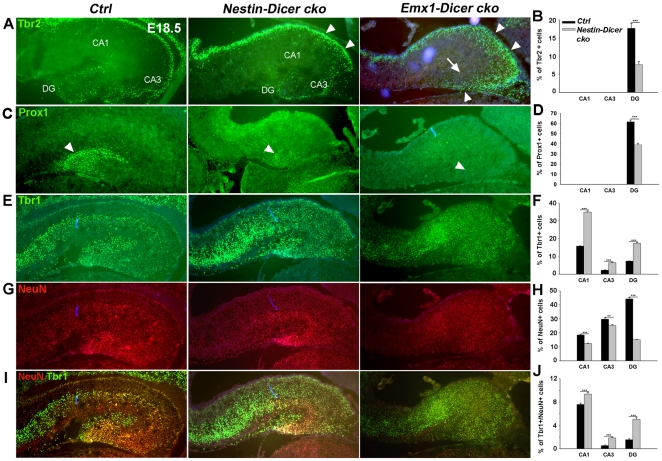
*Dicer* deletion causes early differentiation and abnormal maturation of hippocampal neurons at E18.5. (A, B) Tbr2^+^ cells in the dentate gyrus (DG) were greatly reduced in the E18.5 *Nestin-Dicer cko* hippocampus compared to controls (*Ctrl*). Tbr2 expression was undetectable in the DG of E18.5 *Emx1-Dicer cko* mice due to a great reduction of the hippocampus (arrow). More Tbr2^+^ cells were detected in the cortical progenitor zone (arrowheads) in the *Emx1-Dicer cko* and *Nestin-Dicer cko* brains, and in the dentate migratory stream (arrowheads) in the E18.5 *Emx1-Dicer cko* hippocampus. n>3, ***, *P<0.001*. (C, D) Prox1^+^ cells, detected in the DG (arrowheads), were reduced in the E18.5 *Nestin-Dicer cko* hippocampus and undetectable in the *Emx1-Dicer cko* hippocampus. n>3, ***, *P<0.001*. (E, G, I) Early born neurons labeled with Tbr1 were increased, while mature neurons labeled with NeuN were decreased in the CA1, CA3 and DG regions in the E18.5 *Nestin-Dicer cko* hippocampus compared to controls. Tbr1 and NeuN expression was undetectable in the E18.5 *Emx1-Dicer cko* hippocampus due to underdeveloped hippocampal region. (F, H, J) Quantification of Tbr1^+^, NeuN^+^ and Tbr1^+^/NeuN^+^ cells in the E18.5 *Nestin-Dicer cko* and control hippocampus. n>3, **, *P<0.01*; ***, *P<0.001*. At least three *Nestin-Dicer cko* and three control animals were used for all statistical analysis.

To further reveal the underlying mechanisms that cause abnormal hippocampal development in *Dicer* conditional knockout mice, we examined neuronal production. In the E18.5 hippocampus, Tbr2 expression was mostly detected in the DG region, which is likely in progenitors. Tbr2^+^ cells were greatly reduced in the E18.5 *Nestin-Dicer cko* hippocampus (Fig. 7A, B). At the same stage, the DG region was undetectable in the *Emx1-Dicer cko* hippocampus, indicating a severe hippocampal defect (Fig. 7A). Ectopic Tbr2^+^ cells were detected in the cortical progenitor zone along the hippocampal region in both *Nestin-Dicer cko* and *Emx1-Dicer cko* brains, and in the dentate migratory stream in the *Emx1-Dicer cko* hippocampus (Fig. 7A). We next examined expression of Prox1, a DG-specific neuronal marker. While a great reduction of Prox1^+^ cells was observed in the *Nestin-Dicer cko* hippocampus, no Prox1^+^ cells were detected in the *Emx1-Dicer cko* hippocampus (Fig. 7C, D).

Interestingly, more early-born neurons labeled by Tbr1 were observed in the E18.5 *Nestin-Dicer cko* hippocampus compared to controls (Fig. 7E, F). In particular, Tbr1^+^ cells were significantly increased in the CA1 and DG regions of the *Nestin-Dicer cko* hippocampus. However, the number of mature neurons labeled by NeuN was greatly reduced in the CA1, CA3 and DG regions, while the number of NeuN^+^/Tbr1^+^ neurons was increased (Fig. 7G-J). Tbr1 and NeuN expression was undetectable in the E18.5 *Emx1-Dicer cko* hippocampus due to underdeveloped hippocampal region (Fig. 7E, G). Ectopic Tbr2 expressing progenitors may contribute to increased Tbr1^+^ neurons in the hippocampus. However, maturation of hippocampal neurons, labeled by Prox1 and NeuN, is reduced. Therefore, our results indicate that while blocking miRNA biogenesis causes early neuronal differentiation, it has a negative effect on maturation of hippocampal neurons.

**Figure 8:**
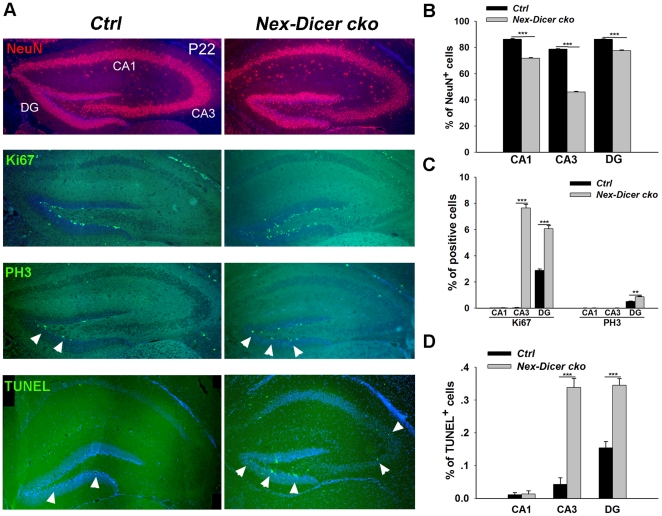
*Dicer* deletion caused defects in hippocampal neurogenesis in the *Nex-Dicer cko* brain at P22. (A) Fewer NeuN^+^ neurons were detected in the CA1, CA3 and dentate gyrus (DG) regions, in particular the CA3 region, of the P22 *Nex-Dicer cko* hippocampus compared to controls (*Ctrl*). However, an increase of Ki67^+^, PH3^+^ and TUNEL^+^ cells were detected in the CA3 and DG regions (arrowheads) but not the CA1 region of the *Nex-Dicer cko* hippocampus. (B-D) Quantification of NeuN^+^, Ki67^+^, PH3^+^ and TUNEL^+^ cells in the P22 *Nex-Dicer cko* and control hippocampus. At least three *Nex-Dicer cko* and three control animals were used for all statistical analysis. n>3, **, *P<0.01*; ***, *P<0.001*.

We next examined hippocampal neurogenesis in P22 *Nex-Dicer cko* brains, in which miRNA biogenesis was blocked at a later developmental stage. Similar to the *Nestin-Dicer cko* hippocampus, the number of NeuN^+^ cells was greatly reduced in the CA1, CA3 and DG regions, in particular, the CA3 region was more severely affected than other regions (Fig. 8A, B). Furthermore, a higher frequency of mispositioned NeuN^+^ cells was observed along the CA1 and CA3 regions of the *Nestin-Dicer cko* hippocampus. Our results indicate that different timings of *Dicer* deletion affect neurogenesis in distinct regions of the hippocampus, with the CA1 and DG more sensitive to an early loss of miRNAs and the CA3 more sensitive to a late loss of miRNAs.

Interestingly, further examination of progenitor cells in the P22 hippocampus of *Nex-Dicer cko* reveals an increase of Ki67^+^ cell numbers in the CA3 and DG regions, and an increase of PH3^+^ cell numbers in the DG region compared to controls (Fig. 8A, C). Moreover, a great increase of apoptotic cells were detected in the CA3 and DG regions in the P22 *Nex-Dicer cko* hippocampus (Fig. 8A, D). Our results suggest that a late loss of miRNAs selectively affects progenitor proliferation and apoptosis in the CA3 and DG regions of the postnatal hippocampus.

## Discussion

**Figure 9:**
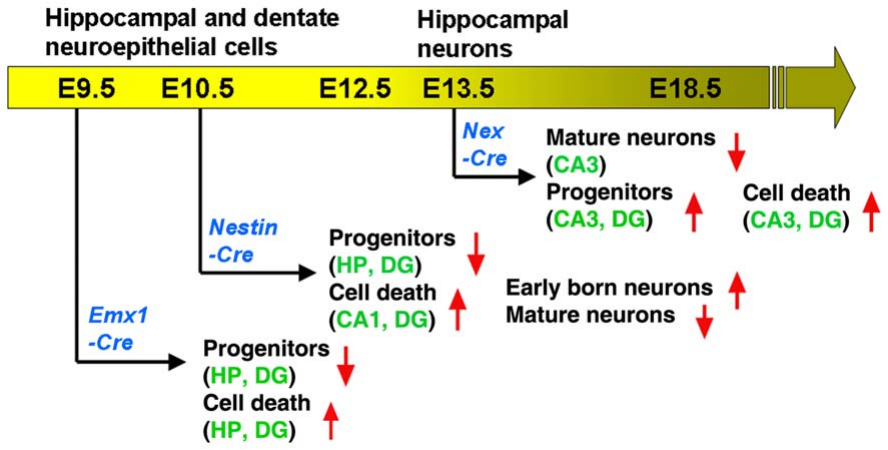
Summary of miRNA functions at different time points of hippocampal development. Dicer ablation using the *Emx1-Cre*, *Nestin-Cre* and *Nex-Cre* lines at different embryonic stages in mice affects progenitor proliferation, cell death and differentiation in the hippocampus (HP) and in different regions in the HP such as CA1, CA3 and dentate gyrus (DG). The up and down arrows point out an increased or decreased effect of miRNAs, respectively.

Using mouse genetic tools, miRNA biogenesis was blocked in the developing hippocampus at different embryonic time points. We have found that early miRNA function is required for proper hippocampal morphogenesis, and that it is essential for continuous expansion of hippocampal neuroepithelial and dentate granular progenitors. Interestingly, we have found that the CA1 and dentate gyrus (DG) regions are more sensitive to an early loss of miRNAs, and the CA3 region is more sensitive to a late loss of miRNAs (Fig. 9). Our studies imply that miRNAs may target distinct genes at different time points to ensure proper embryonic and postnatal hippocampal development and functions.

### Early requirement of Dicer for hippocampal formation

The role of miRNAs has proven to be essential in the development and function of the CNS by ablating *Dicer* using different *Cre* lines [23]–[25], [28]–[30]. In particular, late embryonic deletion of *Dicer* has caused dendritic branching defects of hippocampal neurons and neurodegeneration [28], [29]. These studies indicate the importance of miRNAs, processed by Dicer, in hippocampal formation and functions.

We here have revealed the requirement of miRNA functions in early hippocampal morphogenesis and progenitor expansion. Previous work has demonstrated that patterning molecules, in particular from the cortical hem, play a critical role in early formation of the hippocampus [5], [6]. We have shown that mice with early ablation of *Dicer* using the *Emx1-Cre* line by E9.5 fail to develop detectable CA1, CA3 and DG regions. Moreover, there are altered expressions of early hippocampal markers and ectopic Reelin-expressing Cajal-Retzius cells in the cortex. These suggest that miRNAs may regulate proper expression levels and domains of hippocampal patterning molecules such as Wnt and BMPs and their downstream genes, and participate in the process of early hippocampal morphogenesis and the development of Cajal-Retzius cells derived from the cortical hem.

In addition, our studies have shown reduced numbers of hippocampal neuroepithelial cells and dentate granular progenitors when early *Dicer* expression is deleted by the *Emx1-Cre* and *Nestin-Cre* lines (Fig. 9). Reduced *Lhx2* expression in progenitors may contribute to smaller hippocampus in the *Emx1-Dicer cko* and *Nestin-Dicer cko* hippocampus. Consistent with the role of Dicer in neural stem cell and neural progenitor development, miRNA functions are generally required for expansion of the neural progenitor pool in the cortex and the hippocampus [26], [27]. Although increased cell death was detected in the *Dicer* deficient hippocampus, the reduced hippocampal size was most likely caused by a pronounced proliferation defect of hippocampal progenitors.

The hippocampal defects are more profound in the *Emx1-Dicer cko* brain than the *Nestin-Dicer cko* brain, which is consistent with phenotypes observed in the cerebral cortex [24]. We think the following reasons may explain the difference: 1) the timing difference. The hippocampal development is timing sensitive. Earlier deletion of miRNAs by the *Emx1-Cre* line causes more severe defects in the hippocampus than the *Nestin-Cre* line; 2) the strength of the Cre activity. The Emx1-Cre may have stronger excision effects of Dicer than the Nestin-Cre and causes a rapid ablation of miRNAs.

### Early and late loss of Dicer affects distinct hippocampal regions

Early deletion of *Dicer* using the *Nestin-Cre* line reduces progenitor numbers in all hippocampal regions. However, we have found that apoptotic cells are mostly localized in the CA1 and DG regions. Moreover, the increase in early differentiated hippocampal neurons is more prominent in the CA1 and DG regions than the CA3 region when miRNA biogenesis is blocked by E10.5. These results suggest that early miRNA functions have a higher impact on neurogenesis in the CA1 and DG regions of the hippocampus (Fig. 9). Interestingly, when *Dicer* ablation occurs by E13.5 using the *Nex-Cre* line, the overall hippocampal morphogenesis appears normal. However, we have found that the CA3 region is more severely affected than other regions. Our results are consistent with a previous report showing more severely reduced mature neurons in the CA3 regions when *Dicer* is ablated by the *CaMKII-Cre* line [29].

Our results and others indicate the timing requirement of miRNA functions for the proper development of distinct hippocampal regions. Some miRNAs that are highly expressed in the hippocampus at an early stage, for example by E10.5, perhaps play an important role in the formation of the CA1 and DG regions, while some miRNAs highly expressed by E13.5 may be essential for CA3 region development. These miRNAs may regulate different target genes and ensure proper hippocampal morphogenesis. Moreover, our results also imply that the developmental timings of distinct hippocampal regions are different: the commencement of the CA1 and DG regions is perhaps earlier than the CA3 region.

Moreover, surprisingly, we have found an increase in proliferating and apoptotic cells in the CA3 and DG regions of the postnatal hippocampus in P22 *Nex-Dicer cko* mice, suggesting an active turnover of progenitors in the CA3 and DG regions. Loss of miRNAs at a later stage may release their repression of some target genes that normally promote proliferation and apoptosis, and in turn selectively increases progenitor numbers in the CA3 and DG regions. Interestingly, a recent study has reported that mice with *Dicer* ablation using inducible *CaMKII-Cre* line at adult stages display enhanced learning and memory [30]. NSCs in the adult DG region have the potential to continuously generate new neurons, which is important for learning and memory functions [3], [4]. Concomitantly, late ablation of *Dicer* in adult mice may selectively promote expansion or turnover of NSCs and progenitors in the DG region and contribute to enhanced learning and memory performance [30]. Due to the postnatal lethality of *Nex-Dicer cko* mice by P23 (Hong and Sun, unpublished data), we could not further examine whether more proliferating cells in the CA3 and DG regions are also detectable in the adult *Dicer* knockout hippocampus.

Our findings of time-based functions of miRNAs for hippocampal development indicate the complexity of the miRNA regulation in proliferation, survival and differentiation of hippocampal progenitors (Fig. 9). There is perhaps a group of miRNAs that are highly expressed in the hippocampus at early stages. These miRNAs may play a critical role in patterning the specific domains for the hippocampus and the cortical hem. These miRNAs also function in maintaining proliferation and survival status of hippocampal progenitors. Moreover, a group of miRNAs may be highly expressed in the postnatal hippocampus and play a role in controlling differentiation and maturation of neurons in distinct hippocampal regions such as the CA1 and CA3 region. Identifying specific miRNAs will help reveal miRNA-target networks that are essential for embryonic and postnatal development of the hippocampus. These studies will further shed light on noncoding miRNA functions in cognitive behaviors such as learning and memory.

## Materials and Methods

### Mouse lines and genotyping

The floxed *Dicer* transgenic mice (*Dicer^flox/flox^*) (C57/BL6 x 129 background, provided by the Greg Hannon's lab at the Cold Spring Harbor Laboratory) [36] were bred with three *Cre* lines: *Nestin-Cre* (Stock Number: 003771, C57/BL6 background), *Emx1-Cre* mice (Stock Number: 005628, C57/BL6 background) (The Jackson Laboratory) and *Nex-Cre* (C57/BL6 background, provided by Drs. M. Schwab and K. Nave at Max-Planck-Institute of Experimental Medicine, Goettingen, Germany). Subsequently generated *Dicer* conditional knockout (*cko*) mice were named *Emx1-Dicer cko* (*Dicer^flox/flox^*; *Emx1-Cre*), *Nestin-Dicer cko* (*Dicer^flox/flox^*; *Nestin-Cre*) and *Nex-Dicer cko* (*Dicer^flox/flox^*; *Nex-Cre*).

For staging of embryos, midday of vaginal plug formation was considered embryonic day 0.5 (E0.5), and the first 24 hours after birth was defined as postnatal day 0 (P0).

All animal procedures reported herein were conducted under IACUC protocol (#0807-770A) approved by Weill Cornell Medical College.

### Genotyping of Dicer conditional knockout mice

Mouse tail-tip biopsies were used for genotyping by PCR reactions using the following primer pairs: for *Cre*, 5′-TAAAGATATCTCACGTACTGACGGTG-3′ and 5′-TCTCTGACCAGAGTCATCCTTAGC-3′ (product size: 350 bp); for *Dicer*, 5′-ATTGTTACCAGCGCTTAGAATTCC-3′ and 5′-GTACGTCTACAATTGTCTATG-3′ (product sizes: 767 bp from the *Dicer^flox^* allele and 560 bp from the wild-type *Dicer* gene).

### BrdU Incorporation

To assess proliferation of neural progenitors in the developing hippocampus, one dose of BrdU (50 µg/g body weight) was administrated by intraperitoneal injection to mice half an hour before sacrifice.

### Tissue preparation and immunohistochemistry

Mouse brains were collected at different ages for *Emx1-Dicer cko*, *Nestin-Dicer cko* and *Nex-Dicer cko* mice, and fixed in 4% paraformaldehyde (PFA) in phosphate buffered saline (PBS) at 4°C overnight, followed by incubating in 30% sucrose in PBS. Brain tissues were embedded in OCT and stored at −80°C until use. Brains were sectioned coronally (10 µm/section for immunohistochemistry; 16 µm/section for *in situ* hybridization) with a Leica cryostat (Leica, CM3050 S).

For immunohistochemistry, sections were incubated in heated solution (1 mM EDTA, 5 mM Tris, pH 8.0; 95–100°C) for 15–20 min for antigen recovery, and cooled down for 20-30 min. Before applying antibodies, sections were blocked in 10% normal goat serum (NGS) in PBT (PBS with 0.1% Tween-20) for 1 hour. Sections were incubated with primary antibodies at 4°C overnight and visualized using goat anti-rabbit IgG–Alexa-Fluor-488 and/or goat anti-mouse IgG–Alexa-Fluor-594 (1∶350, Molecular Probes) for 1 hour at room temperature. Images were captured using a Leica digital camera under a fluorescent microscope (Leica DMI6000B). Primary antibodies against the following antigens were used: bromodeoxyuridine (BrdU) (1∶50, DSHB), phospho-histone H3 (PH 3) (1∶1000, Upstate), Ki67 (1∶500, Abcam), Tbr2 (1∶500, Abcam), Prox1 (1∶1000, Chemicon), Tbr1 (1∶500, Abcam) and NeuN (1∶300, Chemicon).

### Nissl staining

Brain sections (10 µm) were processed through incubation in the following solutions in order: ethanol/chloroform (1∶1, overnight), 100% ethanol (30 sec), 95% ethanol (30 sec), distilled water (30 sec, twice), cresyl violet (3–5 min), distilled water (2 min, three times), 50% ethanol (2 min), 95% ethanol (5–30 min), 100% ethanol (5 min, twice), xylene (3 min, twice), and then mounted with a coverslip.

### In situ hybridization

Digoxygenin (DIG)-labeled sense and antisense mRNA probes were produced by *in vitro* transcription. The *in situ* hybridization on sections was performed as described [37]. Briefly, the sections were hybridized at 65°C overnight and washed. After blocking for 2 hours, sections were labeled with anti-DIG antibody (1∶1,500, Roche) at 4°C overnight and washed, stained with BM purple (Roche) at room temperature until ideal intensity. The images of *in situ* hybridization were collected using a Leica digital camera under a dissection scope (Leica, MZ16F).

*In situ* hybridization for microRNA expression was performed according to previously published methods with modifications using locked nucleic acid (LNA) probes [38]. Briefly, after fixation with 4% PFA, acetylation with acetylation buffer (1.33% Triethanolamince, 0.25% Acetic anhydride, 20 mM HCl), treatment of proteinase K (10 mg/ml, IBI Scientific) and pre-hybridization (1×SSC, 50% Formamide, 0.1 mg/ml Salmon Sperm DNA Solution, 1×Denhart, 5 mM EDTA, pH 7.5), brain sections were hybridized with DIG-labeled LNA probes at 45-55°C overnight. After washing with pre-cooled wash buffer (1×SSC, 50% Formamide, 0.1% Tween-20) and 1xMABT, sections were blocked with blocking buffer (1x MABT, 2% Blocking solution, 20% heat-inactivated sheep serum) and incubated with anti-DIG antibody (1∶1,500, Roche) at 4°C overnight. Brain sections were washed with 1xMABT and staining buffer (0.1 M NaCl, 5 mM MgCl_2_, 0.1 M Tris-HCl, pH 9.5), then stained with BM purple (Roche) at room temperature until ideal intensity. The microRNA LNA probes (Exiqon) were 3′ end labeled with DIG–ddUTP with terminal transferase using the DIG–3′ end labeling kit (Roche).

### TUNEL Assay

To identify apoptotic cells in the hippocampus, a TUNEL assay was performed using the Apop Tag Fluorescein *in situ* Apoptosis detection kit (Chemicon) on 10 µm frozen sections according to the manufacturer's instructions.

### Cell counting and statistic analysis

Coronal sections were collected in the medial hippocampal region. At least four sections from each brain were chosen for antibody labeling and TUNEL assay. Positive cells were counted in a field, a randomly selected view in the hippocampus. For instance, the hippocampal neuroepithelium (HN) and dentate gyrus (DG) in the E15.5 hippocampus, and the CA1, CA3 and DG regions in the E18.5 and P22 hippocampus were analyzed. At least 5 fields were selected in each hippocampal region on each section. Positive cells and percentage of positive cells for each marker in distinct hippocampal regions were presented.

At least three *Dicer* conditional knockout and three control littermate animals were used for all statistical analyses. Data were shown as mean ± standard error of the mean (S.E.M). Statistical comparison was made by an analysis of variance (unpaired Student's *t*-Test).
